# Tyrosinase Inhibitor Activity of Coumarin-Resveratrol Hybrids

**DOI:** 10.3390/molecules14072514

**Published:** 2009-07-13

**Authors:** Antonella Fais, Marcella Corda, Benedetta Era, M. Benedetta Fadda, Maria Joao Matos, Elias Quezada, Lourdes Santana, Carmen Picciau, Gianni Podda, Giovanna Delogu

**Affiliations:** 1 Dipartimento Scienze Applicate ai Biosistemi, Università degli Studi di Cagliari, Cittadella Universitaria di Monserrato, S.S. 554 Km 0.700 bivio per Sestu, 09042 Monserrato, CA, Italy; E-mails: corda@unica.it (M.C.), era@unica.it (B.E.), faddam@unica.it (M-B.F.); 2 Departamento de Química Orgánica, Facultade de Farmacia, Universidade de Santiago de Compostela, 15782 Santiago de Compostela, Spain; E-mail: lourdes.santana@usc.es (L.S.); 3 Dipartimento Farmaco Chimico Tecnologico, Università degli Studi di Cagliari, Via Ospedale 72, 09124 Cagliari, CA, Italy; E-mails: carmenpicciau@alice.it (C.P.), gpodda@unica.it (G.P.), mariacmatos@gmail.com (M-J.M), elias.quezada@usc.es (E.Q.)

**Keywords:** tyrosinase inhibitors, resveratrol, coumarins

## Abstract

In the present work we report on the contribution of the coumarin moiety to tyrosinase inhibition. Coumarin-resveratrol hybrids **1-8** have been resynthesized to investigate the structure-activity relationships and the IC_50_ values of these compounds were measured. The results showed that these compounds exhibited tyrosinase inhibitory activity. Compound 3-(3’,4’,5’-trihydroxyphenyl)-6,8-dihydroxycoumarin (**8**) is the most potent compound (0.27 mM), more so than umbelliferone (0.42 mM), used as reference compound. The kinetic studies revealed that compound **8** caused non-competitive tyrosinase inhibition.

## Introduction

Tyrosinase (EC 1.14.18.1) is an enzyme with a dinuclear copper centre, widely distributed in Nature and mainly involved in the formation of pigments such as melanins and other polyphenolic compounds [1]. Roughly speaking the tyrosinases consist of three domains, of which the central domain contains the Cu-binding sites, called CuA and CuB. In this way their overall design is similar to that of arthropod hemocyanins. Matoba and coworkers [2] established the crystallographic structure of tyrosinase, enabling a better understanding of its mechanism of action. This enzyme uses molecular oxygen to catalyze the oxidation of monophenols to their corresponding *o*-diphenols (monophenolase or cresolase activity) and their subsequent oxidation to *o*-quinones (diphenolase or cathecolase activity). The active centre of tyrosinases , formed by dinuclear coppers, is flexible during catalysis.

Within different taxa, the sequence homology of tyrosinases is high and conserved domains can be identified [3]. The enzyme seems to be almost universally distributed in animals, plants, fungi and bacteria but much is still unknown about its biological function. In higher plants, it protects the plant against insects and microorganisms, catalyzing the formation of a melanin scab impervious to further attack [4,5]. Moreover, tyrosinase is responsible of browning of certain fruits and vegetable during their handling, processing and storage after harvest, and both browning of fruit and skin darkening can be suppressed, at least partially, by deactivation of tyrosinase. In mammals, the enzyme is responsible for skin pigmentation [6,7] and is linked to Parkinson disease and other neurodegenerative diseases [8,9].

The study of tyrosinase inhibitory activity became of interest in recent years because of the significant industrial and economic impact of the inhibitors of this protein. Recently, different inhibitory compounds derived from natural sources or partly/fully synthetic have been tested [10]. Studies on tyrosinases, their substrates and inhibitors, are needed to better understand the details of its biological activity and to know how to control it [11].

Our research aimed to study compounds that hypothetically would be good tyrosinase inhibitors, by using all available structural information. Our experience on both coumarin and resveratrol structures, it has encouraged us to examine coumarin-resveratrol hybrids in this context.

Resveratrol (3,5,4’-trihydroxy-*trans*-stilbene) is a polyphenolic phytoalexin produced in plants via a metabolic sequence induced in response to biotic or abiotic stress factors. It has been found in a multitude of dietary plants and derived products, such grapes, peanuts, legumes and in red wine. It has been shown to be an antimutagen, antioxidant, and inducer of quinone reductase. In addition, many biological effects have been attributed to resveratrol, such as modulation of hepatic apolipoprotein, activation of the estrogens’ receptor, inhibition of platelet aggregation and *in vitro* eicosanoid synthesis, inhibition of transcription and enzyme activity of cyclooxygenase-2 [12]. All these effects point toward the possibility that resveratrol may contribute to the cardioprotective effects offered by red wine consumption [13,14,15].

Inhibitory effects of hydroxystilbene compounds on mushroom tyrosinase activity have been evaluated [16] and some of these have significant inhibitory activity. In this group, resveratrol, better known for its cancer-chemopreventive potential [17], represents the simplest structure, with free hydroxyl groups at positions 3, 5 and 4’. This compound showed stronger DOPA oxidase inhibitory activity than kojic acid [18].

On the other hand, coumarins represent a class of compounds of interest for a long time due to their biological activities: they have been shown to be useful as antitumoural and anti-HIV agents [19]. In addition, they have show to possess cardioprotective properties: many of them are selective coronary vasodilators, an effect that may be related to a Ca^2+^-antagonistic activity [20]. In addition, Masamoto *et al*. [21] investigated the structure-activity relationship of 18 coumarins for their inhibitory activity on mushroom tyrosinase, and they find that esculetin exhibited the strongest inhibitory activity. Recently, in contrast with the findings of Masamoto, Sollai *et al*. [22] have shown that esculetin is nevertheless to be considered a tyrosinase substrate rather than an inhibitor, whereas umbelliferone appears to be an inhibitor of the mentioned oxidase.

Based on this information, and considering the importance of hydroxyl groups in tyrosinase inhibitory activity [23,24], we synthesized the hydroxylated coumarin-resveratrol hybrids **1**-**8** in order to study their inhibition mechanisms to provide a basis for the development of new effective tyrosinase inhibitors.

## Results and Discussion

**Scheme 1:**
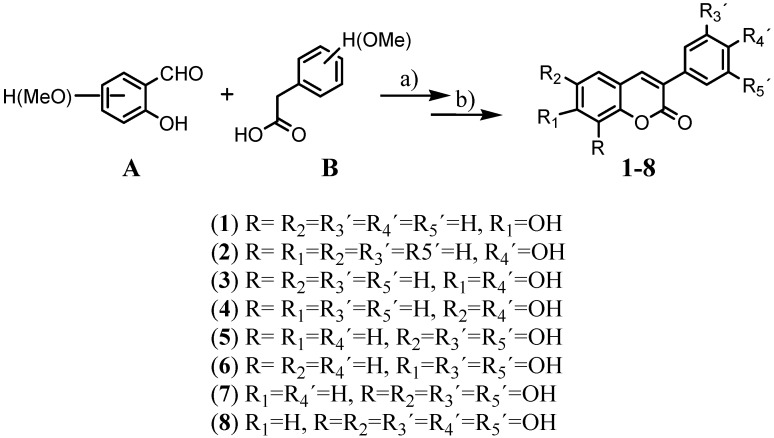
General synthetic route to obtain compounds **1**-**8.**

The studied compounds **1-8** were prepared by the reaction of methoxy substituted *o*-hydroxy-benzaldehydes with the corresponding arylacetic acids, according to the Perkin conditions (Scheme 1) [25,26,27,28].

*Reagents and conditions*: a) DCC, DMSO, reflux; b) HI, Ac_2_O, AcOH, reflux.

Inhibitory effects on L-3,4-dihydroxyphenylalanine (L-DOPA) oxidizing diphenolase activity of tyrosinase of the hydroxyphenyl coumarin derivates and umbelliferone are summarized in Table 1. Compound **1** is an umbelliferone analogue with one phenyl group at C3. The phenyl substituent led to a dramatic loss of potency, and compound **1** did not inhibit any tyrosinase activity. This result indicates that 3-phenyl substituent of umbelliferone may be an important factor for the absence of tyrosinase inhibition activity.

Having identified the importance of the 4’-hydroxyl group of flavonoid compounds in the pharmacological activity, we studied the role of hydroxyl group at position 4’ of the coumarin-resveratrol hybrid **2**. Furthermore, we decided to examine the effect of adding hydroxyl groups at the 4’ position of compound **1** (thus obtaining compound **3**). As shown in Table 1, these compounds have no demonstrable inhibitory effects. On the other hand, the inhibitory potency was enhanced in 6-hydroxy-3-(4’-hydroxyphenyl)coumarin (**4**), suggesting that a different position of hydroxyl group substituent of the A ring might be advantageous.

**Table 1:** Inhibitory effect of hydroxyphenylcoumarins derivate on mushroom tyrosinase activity (substrate: L-DOPA).

Compound	% inhibition (at 0.8 mM)	IC_50_ (mM)
**1**	0	>0.1
**2**	0	>0.1
**3**	9.6	>0.1
**4**	26.7	1.56
**5**	0	>0.1
**6**	19.3	3.68
**7**	0	>0.1
**8**	68.3	0.27
**Umbelliferone ^a^**	-	0.42

^a^ Obtained from data in ref. [20].

The coumarin-resveratrol hybrids **5-8** with hydroxyl groups in positions 3’ and 5’ were found to exhibit different inhibitory potencies, depending on the position and number of hydroxyl groups in the A and B rings. The activities of the compounds **5**-**7** did not provide interesting results as none of them showed any significant inhibitory activity (Table 1), but compound **8** showed the highest tyrosinase inhibitory activity (IC_50_=0.27 mM). This could be due to a synergistic effect of the hydroxyl groups on the aromatic rings. This implies that the number of free OH groups plays an important role in determining the activity. It is unclear, however, why the additive effect of the 4’-hydroxyl is different from that of the other hydroxyl substitution patterns, although Gao and co-workers have reported the inhibitory activity of 3’,4’,5,5’,7-pentahydroxyflavone that has a similar structure to compound **8** [29]. Furthermore, under similar experimental conditions, these molecules have shown similar rates of inhibition.

**Figure 1:**
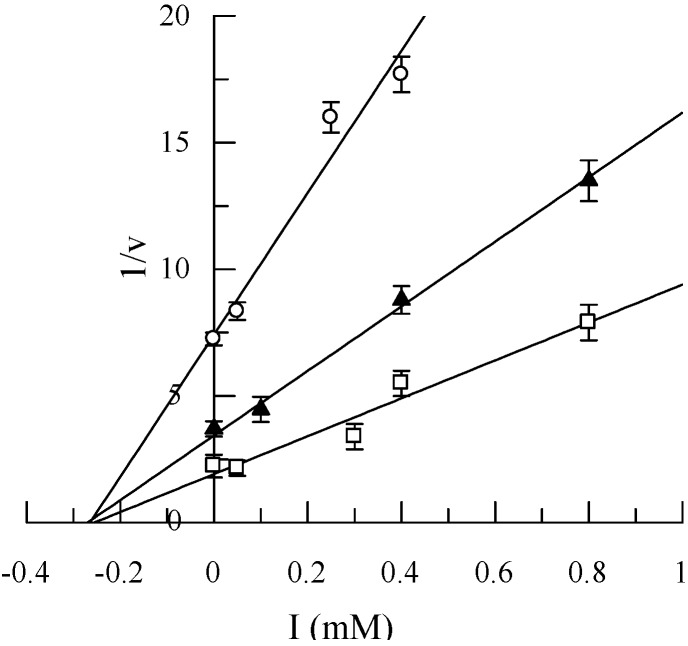
Dixon plot for the inhibition of tyrosinase by compound **8** with respect L-DOPA as substrate at concentrations: (□) 0.5 mM;(▲) 0.25 mM; (○) and 0.1 mM.

To better understand tyrosinase inhibition by compound **8**, we studied its effect on the diphenolase activities of the enzyme. A plot of 1/V vs inhibitor concentration (Figure 1) shows that **8** produced a non-competitive inhibition. In the non–competitive type of inhibition, the inhibitor does not affect the enzyme-substrate combination but affects only the velocity of reaction. The inhibitory activity exerted by **8** is different from that shown from pentahydroxyflavone, which was previously reported as competitive inhibitor [29].

## Experimental

### Chemistry

The studied compounds are all known and were prepared by a traditional Perkin reaction carried out in refluxing dimethylsulfoxide (DMSO) between *o*-hydroxybenzaldehydes (or their methoxy substituted derivatives) **A**, and the corresponding arylacetic acids **B**, using dicyclohexylcarbodiimide (DCC) as dehydrating agent (Scheme 1) [25,26,27]. Hydrolysis of methoxy groups, by treatment with HI in acetic acid/acetic anhydride, gives the desired hydroxyl derivatives **1-8** [25,26,27,28].

### Tyrosinase inhibition assay

Tyrosinase activity assays were performed with L-DOPA as substrate, as previously described [30] with slight modifications. The pre-incubation with enzyme consisted of a 1/15 M phosphoric acid buffer solution (pH 6.8, 1.8 mL), an aqueous solution of mushroom tyrosinase (1000 U/mL, Sigma Chemical Co., 0.1 mL) and DMSO (0.1 mL) with or without an added sample. The mixture was pre-incubated at 25°C for 10 minutes. Then, a 1.5 mM L-DOPA solution (1 mL) was added and the reaction was monitored at 475 nm for 5 min. The detected absorbance increases accompanying the oxidation of the substrate (L-DOPA). The final concentration of dopaquinone was measured using the molar absorption coefficient of 3,700 M^-1^ cm^-1^ [31]. One unit of enzymatic activity was defined as the amount of enzyme transforming 1 μmole of L-DOPA per minute. The percentage of inhibition of tyrosinase activity was calculated as inhibition (%) = (A-B)/A×100, where A represents the difference in the absorbance of control sample between an incubation time of 0.5 and 1.0 min, and B represents the difference in absorbance of the test sample between an incubation time of 0.5 and 1.0 min. The activity of mushroom tyrosinase was determinate by spectrophotometric techniques (Varian Cary 50). DMSO inhibitory activity, in the same experimental conditions, was evaluated as 10.7%.

## Conclusions

Inhibitory effects of hydroxylated coumarin-resveratrol hybrids **1**-**8** on mushroom tyrosinase activity have been evaluated. The experimental data showed that compound **8** possesses the highest activity among the tested hybrids, and it was a non-competitive inhibition with respect to L-DOPA. This result indicates that the number and the position of free OH groups play an important role in determining the activity. Further experiments are in progress in order to know how these compounds interact with the enzyme on molecular basis. Moreover, since the initial step in melanin synthesis is also the same in catecholamine synthesis, we are beginning to study a possible close relationship of inhibitory tyrosinase activity and vasorelaxant activity.
